# Verifying Negative Sentences

**DOI:** 10.1007/s10936-021-09798-9

**Published:** 2021-08-28

**Authors:** Shenshen Wang, Chao Sun, Ye Tian, Richard Breheny

**Affiliations:** 1 Division of Psychology and Language Sciences, University College London, London, UK; 2 Leibniz-Centre General Linguistics (ZAS), Berlin, Germany; 3 MediaTek, Cambridge, UK

**Keywords:** Negation, Sentence verification, Context and relevance, Incremental processing

## Abstract

In the long history of psycholinguistic research on verifying negative sentences, an often-reported finding is that participants take longer to correctly judge negative sentences true than false, while being faster to judge their positive counterparts true (e.g. Clark & Chase, Cogn Psychol 3(3):472−517, 1972; Carpenter & Just, Psychol Rev 82(1):45–73, 1975). While many linguists and psycholinguists have strongly advocated the idea that the costs and complexity of negation can be explained by appeal to context, context-based approaches have not been able to provide a satisfying account of this polarity*truth-value interaction. By contrast, the alternative theory of negation processing, which says that negation is processed by separately representing the positive, does provide a plausible account. Our proposals provide a means for reconciliation between the two views since we argue that negation is a strong cue to a positive context. Here we present our account of why and when negation is often apparently processed via the positive. We review many of the factors that are seen to be at play in sentence verification involving negation. We present evidence that participants’ adoption of the positive-first procedure in sentence-picture verification tasks is conditioned by context.

## Introduction

### Two Procedures for Verifying Negative Sentences

Consider a well-known sentence-picture verification (SPV) task in which participants read sentences like, ‘The star is above the cross’ and are shown a picture containing a star and a cross (Clark & Chase, 1972; Macleod et al., 1978; Mathews et al., 1980; Tversky, 1975). In this task, pictures are always arranged so that one of the mentioned symbols is above the other and it could be such that the arrangement corresponds with that mentioned in the sentence (*/ +), or not (+ /*). The same two pictures can be paired with the negative version of this sentence, ‘The star is not above the cross’. As for virtually all SPV tasks, participants take longer to correctly judge an affirmative (positive) sentence False than True (TA < FA). When it comes to negative sentences in this task, some studies participants take longer to correctly make True judgements than False (FN < TN). However, in other variations of this task (with these sentences and images), it has been found that participants are faster to respond True, regardless of whether the sentence is positive or negative (TA < FA; TN < FN). When it comes to studies with different stimuli to these, we can find both patterns of results for negative sentences (see Trabasso et al., 1971; Young & Chase, 1971; Carpenter & Just, 1975; among others). Additionally, some studies report a polarity*truth-value interaction but without a clear difference between TN and FN (TA < FA; TN = FN) (Wason, 1961; Gough, 1965, 1966; Macleod et al., 1978; Mathews et al., 1980; Trabasso et al., 1971; among others).

It has become relatively clear why, in general terms, both the FN < TN and TN < FN patterns are reported (Clark, 1974). An insight dating from at least Wason (1961), Gough (1965) and others^1^ is that when a participant is faced with a negative sentence, [not[S]], the task can be performed by a process which first evaluates the positive argument of negation, [S], and then reverse the response. Since we know that TA < FA, we know that the argument of a True negative will take longer to evaluate than that of a False negative. If we assume that reversing response requires the same resources whether from True to False or vice versa, then we can see why participants’ responses yield the FN < TN pattern. On the other hand, the task may be performed by a process which computes the states of affairs which make the negative sentence true. Thus, rather as in the Affirmative trials, [not[S]] can be directly evaluated against the image. Assuming that this process takes longer when the sentence is False, we can understand how participants’ responses can yield the TN < FN pattern. Let us refer to the first processing strategy the two-step procedure and the second the one-step, or ‘direct interpretation’ procedure. When a study yields a polarity*truth-value interaction but no clear difference for negative sentences (TN = FN), we can assume that this is the result of both kinds of procedure being employed for negative sentences.

Discussion to date on when and why one procedure is preferred over another has yielded several answers. One clear block on the one-step procedure has to do with the relative ease to compute the actual states of affairs for the negative sentence. For example, ‘the coat is not red’ could be true because the coat is any number of colours, while ‘the door is not closed’ corresponds only to a state of affairs where the door is open. To the extent that SPV tasks require matching a state of affairs for the sentence with that state of affairs depicted in the image, having multiple states of affairs to match from is likely to result in preference for the two-step procedure. This account finds support in the results of Mayo et al. (2004), who report a consistency judgement task that manipulated whether the negative statement had a single verifying state of affairs (‘bi-polar negations’) or several (‘uni-polar’). Their results indicate that participants attend to the positive argument of negation in order to complete the task, but only in the unipolar case, not the bipolar. Similar findings are reported in Orenes et al. (2014). Explicit exploration of this factor for the SPV task is reported in Kroll and Corrigan (1981) who show that in a task which uses the stimuli from Clark and Chase (1972) discussed above, certain filler items confounded the expectation that the mentioned objects only appear one on top of the other, by having the symbols appear side-by-side. In the original study, ‘A is not above B’ is bipolar in that only one state of affairs (B above A) makes it true; in the new condition, this bipolarity assumption is removed and the result is that fewer participants adopt a one-step procedure.

Another factor that has been explored is the timing of presentation of sentence and image. Clark (1974) reports how presentation of the components of a SPV task stimulus can affect the degree to which participants adopt one type of procedure over another. This is demonstrated clearly in Tversky (1975) which uses Hebrew versions of stimuli from Clark and Chase (1972). In one study, sentence and picture are presented at the same time (the procedure followed in Clark & Chase, 1972) and this led to a polarity*truth-value interaction—indicating the use of two-step procedure among participants. In a second study, sentences are presented five seconds prior to the image and here only a main effect of truth-value was found (TA = TN < FA = FN). It seems clear in this study that long exposure to the sentence prior to image allows participants time to infer the truth-making state of affairs regardless of polarity. In related work, Matthews et al. (1980, Experiment 1) also present sentence separately before image but in a self-paced procedure, allowing reading-time and verification-time each to be recorded. Analyses of verification times showed two groupings of participants according to response patterns. One group took longer to respond to negative sentences and also displayed a polarity*truth-value interaction typical of the two-step procedure. The other group showed the pattern found in Tversky (1975) with no effect of polarity and only a main effect of truth-value. Overall, the verification times of this second group were much lower than the first. Examination of reading times reveals insight into these groupings. The second group took much more time reading negative sentences than the first. It seems clear that one group is taking time to compute the truth-making state of affairs and use this information to perform the task when presented with the image. I.e. they use the one-step procedure. The shorter reading time goes with the two-step procedure, indicating a more superficial processing of the sentence which leaves the meaning of negation un-incorporated into the semantic interpretation. That the two-step group take longer at the verification stage is consistent with the idea that participants evaluate the argument of negation (*[S]*) against the image and then have to reverse the response. In fact, detailed analysis by Clark and Chase (1972) and Carpenter and Just (1975) obtains estimates of this ‘reversal’ time, supporting the idea that it is constant across truth values.

It is perhaps unsurprising that participants who infer the actual, described states of affairs for negative sentences take longer to read a sentence than those who do not. As Clark and Chase (1972) note, the counterpart of sentential negation in an affirmative sentence would be an operator whose meaning is an identity function. It stands to reason that inferring what states of affairs are truth-makers when considering the result of composing a sentence meaning with identity would typically involve less computational resources compared to when the composition is with negation, because the latter operation requires extra steps of inference. Beyond the reading time differences reported in Matthews et al. (1980), further evidence for this cost of computing the actual state of affairs is reported in Darley, Kent, and Kazanina (2020) and shows up indirectly in individual difference research, such as Reuter et al. (2018), and Margolin (2015); also indirectly in McLeod et al. (1978) and Matthews et al. (1980), who find that participants with better visual abilities are more inclined to adopt the one-step procedure.

As it has been established that two different procedures are commonly implemented by participants in SPV tasks, we can ask what explanations can be given for this fact? In the next section, we outline two broad theoretical approaches to negative sentence comprehension and discuss how each can explain why we find both one-step and two-step procedures.

### Composite vs. Interpretive Theories of Negation Comprehension

Broadly speaking, there are two theoretical approaches to negative sentence comprehension. Several proposals adopt a *composite approach* to negative sentence comprehension. According to this approach, the process of representing an interpretation of a sentence with negation (‘the dots are not red’) is composed of parts which reflect what we see at the level of linguistics structure—negation and its argument. Some versions of this approach simply assume that negation is represented at an amodal conceptual level (NOT(RED(DOTS))) as in Clark and Chase (1972), Carpenter and Just (1975) and elsewhere. Alternatively, simulationist approaches have been offered which are based on the idea that multiple representations are created, which reflect the representation of the positive argument (red dots) and its subsequent rejection, as in Kaup et al., (2005, 2006, 2007a).

A second approach is more like a null hypothesis approach and could be labelled *interpretive*. It is based on the idea that any representation which may be constructed in the course of comprehending ‘[not[S]]’ uses the truth-functional meaning of negation to make inferences that are relevant in the context. Importantly, it is not necessary, according to an interpretive view, that these mental representations separately involve the positive argument of negation. For example, take a situation where it is known that either John is at home or he is at the movies; and the speaker utters the sentence, “John is not at the movies”, then the audience may infer that John is at home; and this may be the focus of the audience’s attention and hence what becomes mentally represented. A more simple example might be hearing, “The window is not open” and representing the window as being shut. The interpretive approach is consistent with ideas from psycholinguistics that language processing is incremental, where comprehension processes are geared toward representing aspects of what is the case, given the truth of the sentence (Tanenhaus et al., 1976).

When it comes to providing an account of experimental results, researchers who favour a composite approach to sentential negation have pointed to the fact that many participants approach SPV tasks using a two-step procedure. The ready adoption of this procedure provides suggestive evidence for the active representation of the argument of negation in the process of correctly evaluating a negative sentence; and this is taken as evidence that comprehension processes do yield composite structures. Further support for the composite view can be found in evidence from a wide range of paradigms beyond SPV tasks, which indicate active representation of the positive argument of negation. These include probe recognition (Hasson & Glucksberg, 2006; Kaup et al, 2007a), ERP (Dudschig et al., 2019; Fischler et al., 1983), visual world (Nordmeyer & Frank, 2014; Orenes et al., 2014) and mouse-tracking (Dale & Duran, 2011; Darley et al., 2020).

One challenge for composite accounts comes from results, some of which are mentioned above, which do not show evidence of participants exploiting representations of the positive argument in performing SPV tasks (one-step procedure). Evidence also comes from probe recognition studies (Tian et al., 2010); ERP (Nieuwland & Kuperberg, 2008), visual world (Tian et al., 2016) and mouse-tracking (Dale & Duran, 2011) among other paradigms. However, composite theorists have argued that these results rely either on participant strategies, such as translating a negative sentence into a positive, or creating mental images for the sentence (Clark, 1974; Matthews et al., 1980) or on the fact that a first step in a two-step procedure is not detected due to delay (Kaup et al., 2007b). The challenge for any account of negation is to explain why, across so many paradigms, participants often exploit the positive argument in the task, and composite accounts can at least provide an answer. When it comes to SPV tasks, the specific challenge for interpretivist approaches is to explain how representations of the positive argument emerge at all, when the account is that the normal mode of language comprehension is to infer what is the case, given the truth of the sentence.

### A Role for the Source of Relevance in Models of Incremental Language Processing.

A separate tradition in negation research has been to explain particular effects in terms of the pragmatics of negation and the role that context plays in sentence processing (Horn, 1989; Nieuwland & Kuperberg, 2008; Wason, 1965). Here we want to develop an interpretivist approach to SPV tasks by following specific theoretical proposals in this tradition, which are outlined in Tian et al., (2010, 2016) and Tian and Breheny (2016, 2019). We argue that sentence comprehension processes are part of a cognitive system which is geared toward making inferences about speech acts, in which a social actor (the speaker) produces a stimulus with a particular purpose. According to most contemporary approaches which explain how the content of an informative speaker’s message is derived, a context must also be inferred in which the message would achieve relevance (Roberts, 1996; Sperber & Wilson, 1986; Stalnaker, 1978). Our proposal is that incremental and probabilistic language processes have two simultaneous aims: to compute the sentence content and the intended Source of Relevance (SoR).^2^ Language processes thus exploit information in the linguistic stimulus, in addition to any contextual information, to infer *both* sentence content *and* SoR. The idea that language processes relate the current utterance to context in this way gains support from work on syntactic parsing (Clifton & Frazier, 2012, 2016) and pronoun resolution (Kehler & Rohde, 2016).

In the case of processing negative sentences, our claim is that, when presented in the absence of other information, sentential negation is a strong cue to a specific class of SoRs, in which the positive argument is a live possibility which the speaker intends to exclude. Here we refer to this kind of SoR as the *Default* context. Thus, if language processes infer a default context, attention can be drawn to the positive state of affairs. However, the presence of other cues, such as prosodic information or a preceding question can override this default. Our account finds support in probe-response and visual world paradigms (Tian et al., 2010; 2016). Here we extend this account to sentence-picture verification: In Default contexts, attention may be drawn to the positive state of affairs and this may both interfere with a 1-step verification procedure and result in the adoption of the 2-step procedure. As noted above, there is evidence that inferring states of affairs supporting the positive may take less time than inferring states of affairs that actually make the negative sentence true. Thus default contexts in which negation is uni-polar (Carpenter & Just, 1975; Kaup et al., 2007a; Mayo et al., 2004; Orenes et al., 2014), or only contextually bipolar (Clark & Chase, 1972) should see more interference from the positive, since inferring context would be less costly than content in these cases (Autry & Levine, 2014).

#### The Current Studies

As discussed above, SPV tasks with negative sentences elicit two kinds of processing strategy. While composite approaches can point to the use of a two-step procedure as supporting evidence that negation comprehension yields a composite representation, an alternative explanation for the use of a two-step procedure is rooted in the idea that inferring context can draw attention to the positive state of affairs, and this can be exploited in verification tasks. Previous research supports the idea that simple negative sentences presented without supporting context can lead to more activation of the positive, but manipulating stimuli in a way that will change likely context, leads to a different outcome (Tian et al., 2010; 2016). Thus, the aim of the current SPV study is to manipulate features of the experimental stimulus to affect the inferred Source of Relevance. To do this, we use context questions. When a speaker directly responds to a positive polar question like, ‘Is the banana peeled?’ then the utterance context is normally one in which the positive state of affairs (the banana being peeled) is being actively entertained (see Tian et al., 2021). However, an utterance can address a wh-question like, ‘Which fruit is not peeled?’ in which the normal presupposition is that some fruit is not peeled. Thus, in a polar question context, participants performing a SPV task for a negative statement ‘the banana is not peeled’ would have their attention drawn to the positive argument of negation. This would interfere with use of one-step procedure and encourage the use of a two-step processing procedure. However, when the context is a wh-question with a negative predicate, attention to the source of relevance would not interfere as much with a one-step procedure. The experimental design of our main study (Experiment 1b) is based on this set of ideas: to present positive and negative statements in the context of a polar question (Default Context) or a wh-question whose predicate’s polarity is congruent with the statement (Congruent Context). We predict more evidence of two-step procedure in Default Context than Congruent contexts. The sentences and visual displays used here were adapted from the two-picture experimental items used in Tian and Breheny (2016; Experiment 2). In that study, sentences (‘the banana is not peeled’) were presented alongside the two-object images used in Experiments 1a,b below and it was found that participants generally adopted a one-step procedure. However, because this previous study used filler items designed to encourage a one-step procedure, we conducted Experiment 1a below to establish a baseline against which we can interpret the results of Experiment 1b. Experiment 1a presents sentence and image without a question context.

## Experiment 1a

### Methods

#### Participants

33 participants (mean age:32, standard deviation:12, range:18–68, 26 females) were recruited from Prolific Academic and were paid £ 0.7 for their participation. All participants were native-English speakers and were naïve to the purpose of the experiment. This experiment was approved by the local research ethics committee. Participants were provided with an electronic version of informed consent before taking part.

### Materials and design

This experiment^3^ has a two-by-two within-participants design. The two independent variables are polarity (positive or negative) and truth value (true or false), which generate four experimental conditions as shown in Table 1. On each trial, participants were presented with a single visual display consisting of the task sentence to the left of a picture of side-by-side objects. The task sentences were simple positive and negative sentences, in the form of “the P is/isn’t Q”. In the accompanying image, both objects can be described by the predicate in the task sentence (e.g. a peeled apple and an unpeeled banana), but only one of these matches the actual state of affairs of the predicate. The task sentence was always displayed on the left and the picture context on the right, and the picture was 600 by 300 pixels (two single-item images side-by-side). In this we follow the procedure in classic SPV experiments and the recommendations in Clark and Chase (1972). All predicates in our experiments are ‘binary’ in the sense that their negation can be associated with a unique state affair (peeled/unpeeled; open/closed; folded/unfolded; etc.). 64 items with binary predicates were constructed and each generated four sentence-picture pairs. One version of each item was assigned to one of four presentation lists. Each list contained 64 experiment items, 16 items per condition.

**Table 1:** Experimental design and example items for Experiment 1a

Polarity	Truth value	Sentence	Display
Affm	True	The apple is peeled	
Neg	False	The apple isn’t peeled	
Affm	False	The apple is peeled	
Neg	True	The apple isn’t peeled	

### Procedure

The experiment was built using PennController for Ibex Farm (Drummond, 2020; Zehr & Schwarz, 2018) and distributed online to participants via Prolific.^4^ Participants were randomly assigned to one of the four lists. In each trial, participants were presented with a single visual presentation which included sentence to the left and image to the right (as described in *Materials and Design*) and they pressed either the left arrow key or right arrow key to answer whether the sentence was true or false. The response keys were counterbalanced. Participants were told that both reaction time and response accuracy were recorded, and they should try to respond as quickly and as accurately as possible. Before starting the experimental trials, each participant completed four practice trials with feedback. No feedback was given in the experimental trials. After participants made a response, the experiment proceeded to the next trial. Two short breaks were included.

### Results

#### Data Treatment and Analysis Methods

2 participants with accuracy less than 80% were removed. For reaction-time analysis, we removed trials with an incorrect response (6.2% of the data). In addition, we removed trials with a reaction time above 7609 ms (based on two standard deviations of the dataset). This further removed 2.5% of the data. All mixed-effect analyses were carried out in R (R Core Team, 2017) using the lme4 package (Bates et al., 2015). Significances of effects were assessed using the ‘lmerTest’ package (Kuznetsova et al., 2013). The two-level factors polarity (affirmative: 0.5, negative: –0.5) and truth value (true: 0.5, false: –0.5) were deviation coded.

### Accuracy

**Fig. 1:**
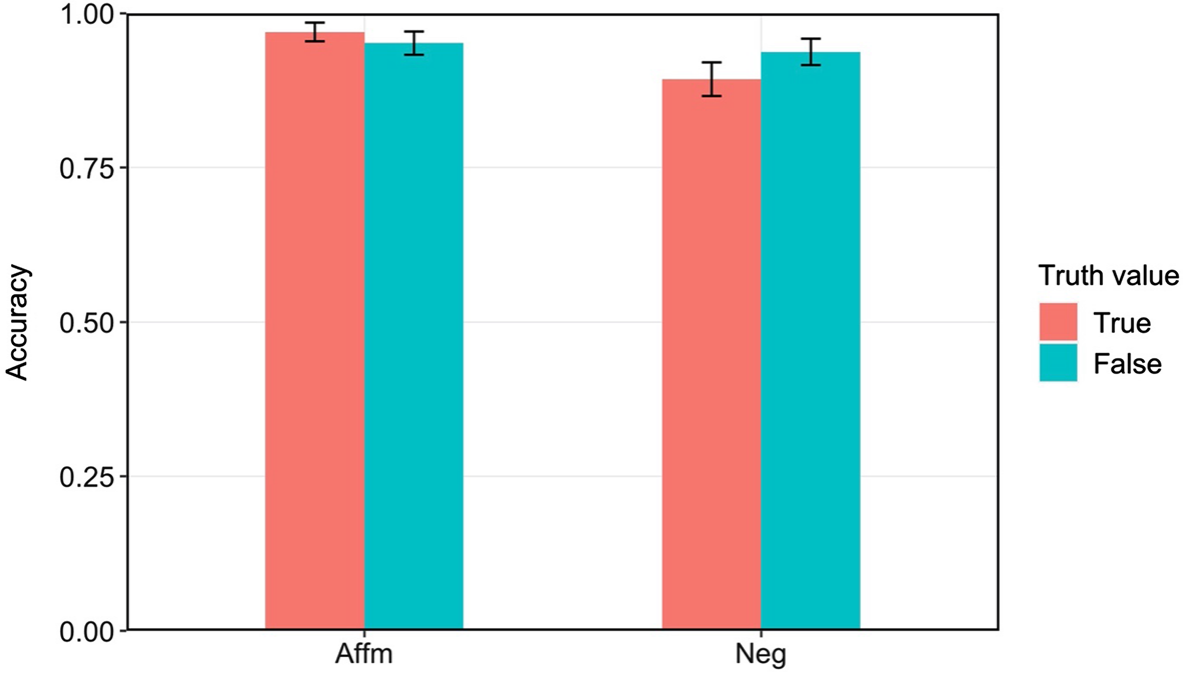
Accuracy for each condition in Experiment 1a. Error bars represent ﻿95% confidence intervals

Figure 1 summarizes the accuracy for each condition. We constructed a generalized mixed effects logistic regression model predicting correctness (correct or incorrect response) from polarity (affirmative or negative), truth value (true or false), and their interaction, including random intercepts and slopes for polarity, truth value, and their interaction for participants and items.^5^ There was a main effect of polarity (β = 0.86, *SE* = 0.22, *p* < 0.001) and a significant interaction between polarity and truth value (β = 1.12, *SE* = 0.43, *p* = 0.009). No effect of truth value was found (*p* = 0.77). The accuracy for TN was significantly lower than FN (β =  − 0.62, *SE* = 0.24, *p* = 0.01), it did not differ significantly between TA and FA (*p* = 0.14).

### Reaction Times

**Fig. 2:**
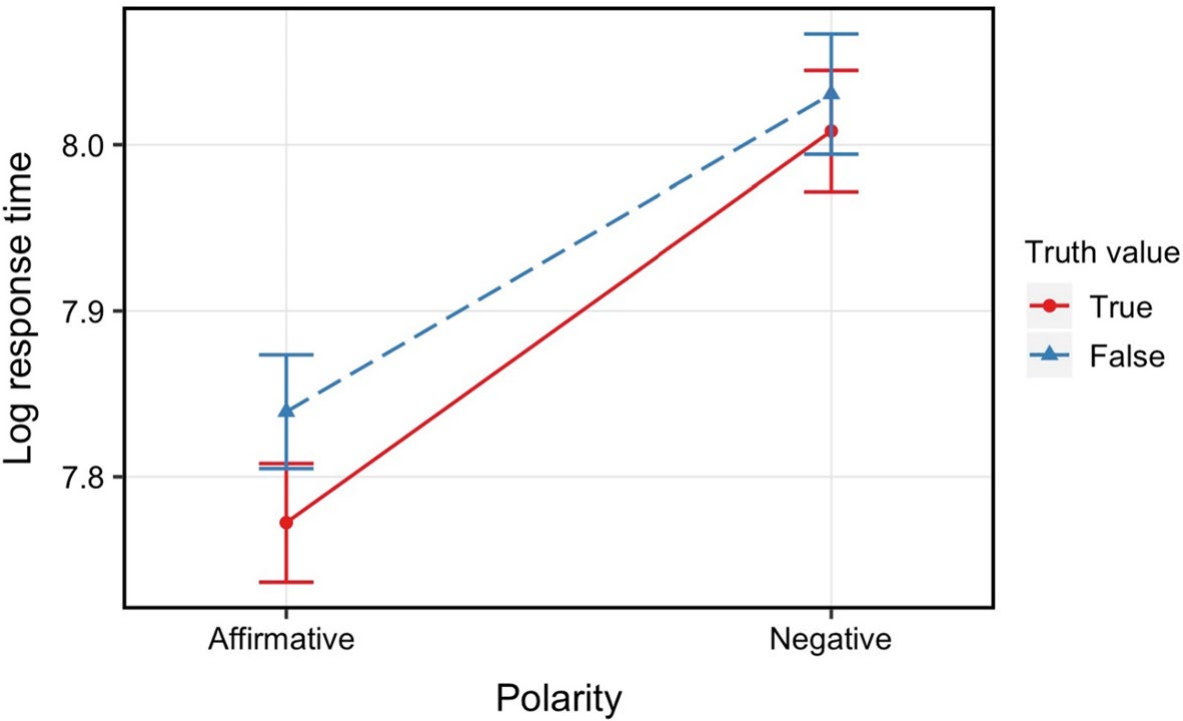
Mean reaction times for each condition in Experiment 1a. Error bars represent ﻿95% confidence intervals

Figure 2 summarizes mean reaction times for each condition. We constructed a linear mixed effects regression model predicting ﻿logarithmised reaction times from polarity (affirmative or negative), truth value (true or false), and their interaction, including random intercepts and slopes for polarity, truth value, and their interaction for participants and items.^6^

The analysis showed main effects of polarity (β =  − 0.22, *SE* = 0.02, *p* < 0.001) and truth value (β =  − 0.05, *SE* = 0.02, *p* = 0.01): verifying negative sentences took longer than verifying positive sentences, and ‘false’ responses took longer than ‘true’ responses. Crucially, we found no significant interaction between polarity and truth value (p = 0.14).

### Discussion

Our results indicate that, overall, the two-step procedure is not highly favoured when our stimuli are presented without prior context. Tian and Breheny (2016) found a similar outcome and attribute it, in part, to the fact that by using natural binary predicates, it is relatively easy for participants to infer the state of affairs which makes the negative sentence true, making the one-step procedure attractive. This contrasts with well-known SPV studies where negative sentences are bi-polar only in context (‘star not above plus’—Clark & Chase, 1972), or not bipolar at all (‘the dots are red’, where dots can be one of three colours—Carpenter & Just, 1975). Moving forward, these results provide us with a baseline against which we can interpret what happens when we manipulate context.

## Experiment 1b

In this experiment, we present the same statement-image pairs as in Exp.1a but in two different contexts: a polar question context and a congruent wh-question context. As discussed above, we predict that the polar question context will result in greater use of the two-step procedure than the congruent context.

### Methods

#### Participants

64 participants were recruited using Prolific and were paid £ 3 for their participation. All participants speak English as a native language and were naïve to the purpose of the experiment. No participants suffered from dyslexia and all had normal or corrected-to-normal vision. This experiment was approved by the local research ethics committee. Participants were provided with an electronic version of informed consent before taking part.

### Materials and Design

This experiment^7^ has a two by two by two within-participants design. The three independent variables are context (default or congruent), polarity (positive or negative) and truth value (true or false), which generate eight experimental conditions as shown in Table 2. In contrast to Experiment 1a, in this case each trial consisted of two separate displays. The first display presented a question, which is either a positive polar question or a wh-question with congruent positive or negative predicates. The second display presented the corresponding answer (i.e. task sentence) to the left of a picture of side-by-side objects. Exactly as in Experiment 1a, both objects can be described by the predicate (e.g. a peeled apple and an unpeeled banana), but only one of which matches the actual state of affairs of the predicate. The task sentences were always in a form that was natural as a response to the question and so were slightly different between the default and congruent contexts. For the default context, the positive polar question “Is the apple peeled?” was answered with “It is” or “It isn’t”. For the congruent context, the wh-question “Which one is/isn’t peeled?” was answered with the noun phrase, “The apple”. Nevertheless, target text in both contexts expresses the same proposition, “The apple is/isn’t peeled”. We adopted this procedure not only because the two-sentence discourse read more naturally, but also because we were concerned that if the entire sentence (‘The apple is/isn’t peeled) were presented instead of the shorter form, then participants would not have to pay attention to the context in order to verify the sentence. The task sentence was always displayed on the left and the picture context on the right, and the picture was 600 by 300 pixels (two single-item images side-by-side).

**Table 2:** Experimental design and example items

Context	Polarity	Truth value	Question	Elided answer	Display
Default	Affm	True	Is the apple peeled?	It is	
Congruent	Affm	True	Which one is peeled?	The apple	
Default	Neg	False	Is the apple peeled?	It isn’t	
Congruent	Neg	False	Which one isn’t peeled?	The apple	
Default	Affm	False	Is the apple peeled?	It is	
Congruent	Affm	False	Which one is peeled?	The apple	
Default	Neg	True	Is the apple peeled?	It isn’t	
Congruent	Neg	True	Which one isn’t peeled?	The apple	

64 items with the same binary predicates and images as in Experiment 1a were constructed. Each item generated four answer-picture displays which were paired with one of two preceding questions. One version of each question/answer-picture pair was assigned to one of eight presentation lists. Each list contained 64 experiment items, 8 items per condition.

### Procedure

**Fig. 3:**
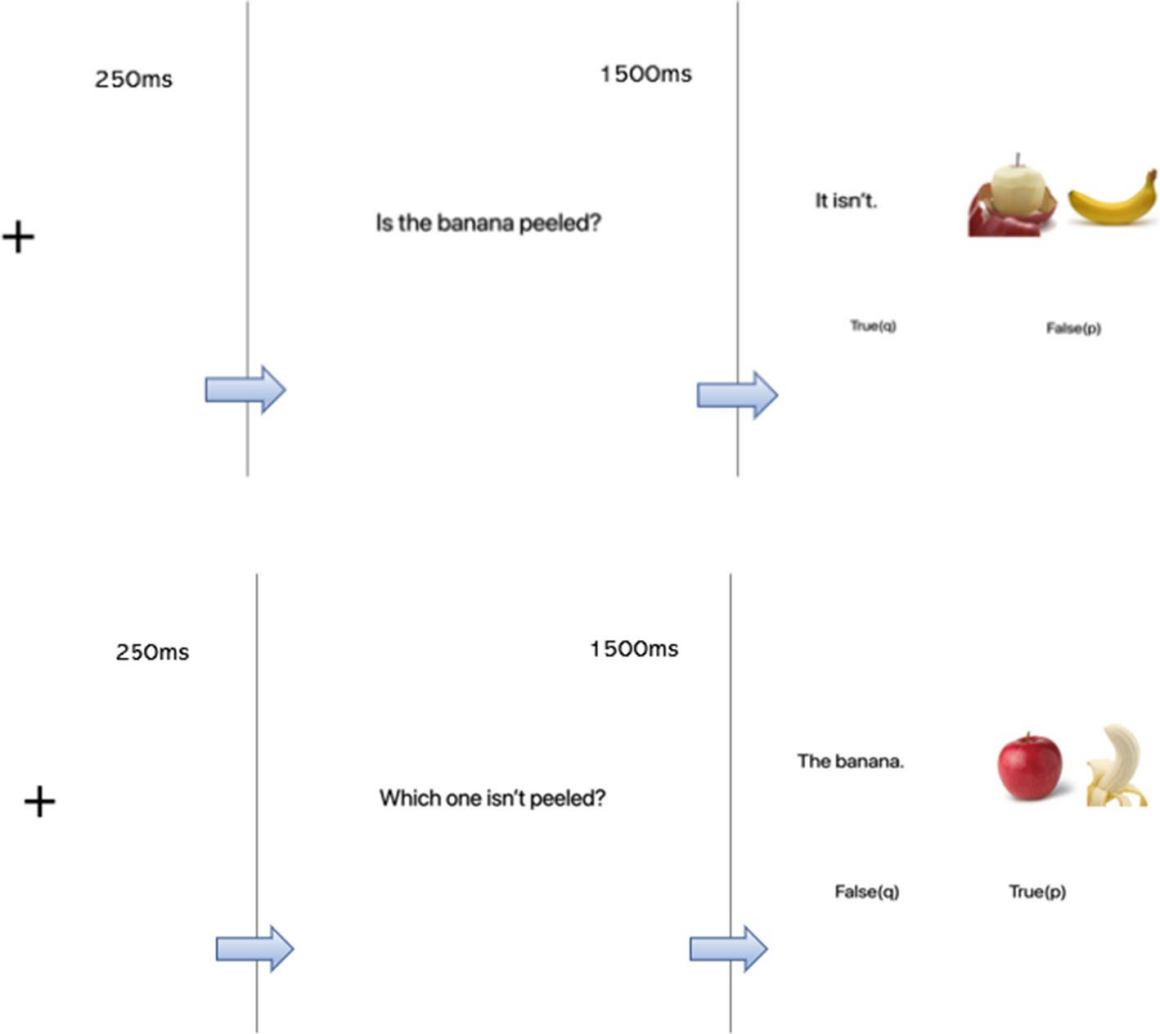
Procedure. An example of Default-Neg-True trial (*top*) and an example of Congruent-Neg-False trial (*bottom*). Context questions appear for 1500 ms prior to target screen

The experiment was created and hosted using the Gorilla Experiment Builder^8^ (www.gorilla.sc) and was distributed online to participants via Prolific. Participants were randomly assigned to one of the eight lists. Each trial began with a centrally located fixation cross appearing for 250 ms before the presentation of the explicit question. The question screen was displayed for 1500 ms before disappearing. Then the screen containing the target answer plus the two-item picture was presented (see Fig. 3). Participants pressed either the “q” or “p” key to answer whether the elided answer was true or false. The response keys were counterbalanced (half the participants pressed “q” for “true” while the other half pressed “p” for true). Participants were instructed to place their left middle finger and right middle finger on the corresponding keys of the keyboards. They were told that both reaction time and response accuracy were recorded, and they should try to respond as quickly and as accurately as possible. After participants made a response, the experiment proceeded to the next trial.

Before starting the experimental trials, each participant completed four practice trials with feedback to familiarize the task procedure. There was no feedback to indicate participants whether they made a correct response in experimental trials. Additionally, there were two short breaks midway through the experiment block after trial 23 and trial 44.

### Results

#### Data treatment and analysis methods

7 participants with accuracy less than 80% were removed. For reaction-time analysis, we removed trials with an incorrect response (7.6% of the data). In addition, we removed trials with a reaction time above 6739 ms (based on two standard deviations of the dataset). This further removed 2.2% of the data. The mixed models contained random intercepts and slopes for all fixed effects for participants and item. The random effect correlations were removed due to convergence issues. The two-level factors context (default: 0.5, congruent: –0.5), polarity (affirmative: 0.5, negative: –0.5) and truth value (true: 0.5, false: –0.5) were deviation coded.

### Accuracy

**Fig. 4:**
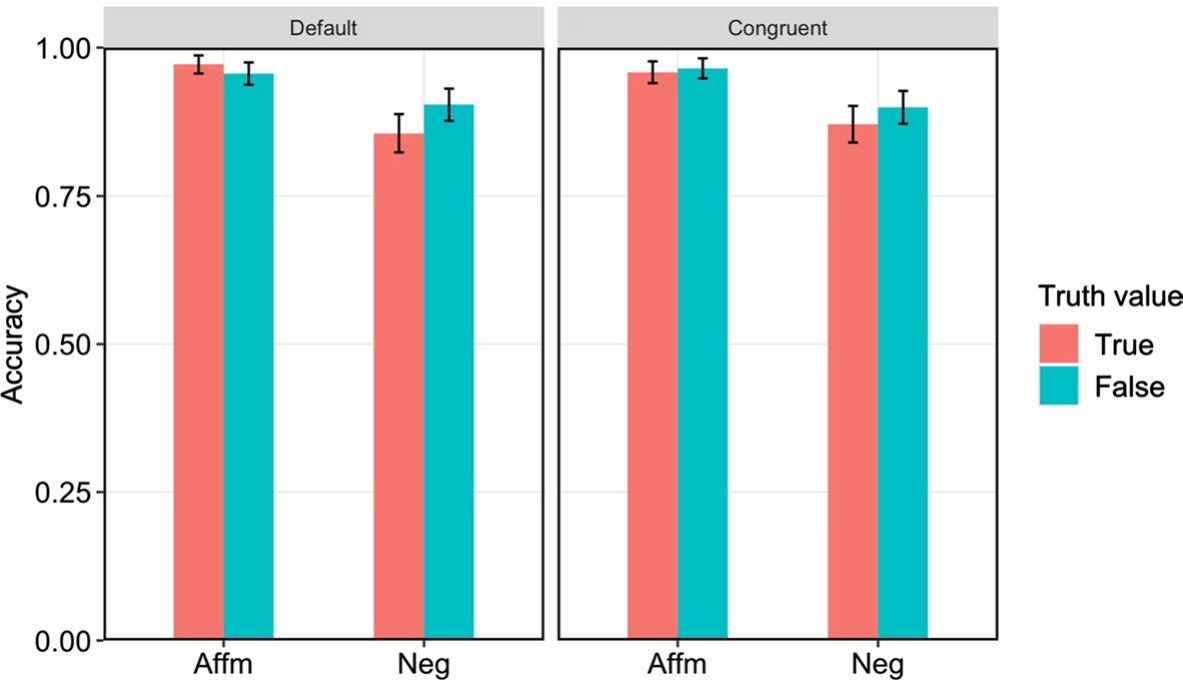
Accuracy for each condition and context. *Error bars* represent 95% confidence intervals

Figure 4 summarizes the accuracy for each condition and context. We constructed a generalized mixed effects logistic regression model predicting correctness (correct or incorrect response) from context (default or congruent), polarity (affirmative or negative), truth value (true or false), and their interaction, including random effects structure as described above.^9^ There was only a main effect of polarity (β = 1.32, *SE* = 0.18, *p* < 0.001), with higher accuracy in verifying affirmative conditions (*M* = 0.96, *SD* = 0.19) compared to negative conditions (*M* = 0.88, *SD* = 0.32). None of the other effects or interactions was significant.

### Reaction times

**Fig. 5:**
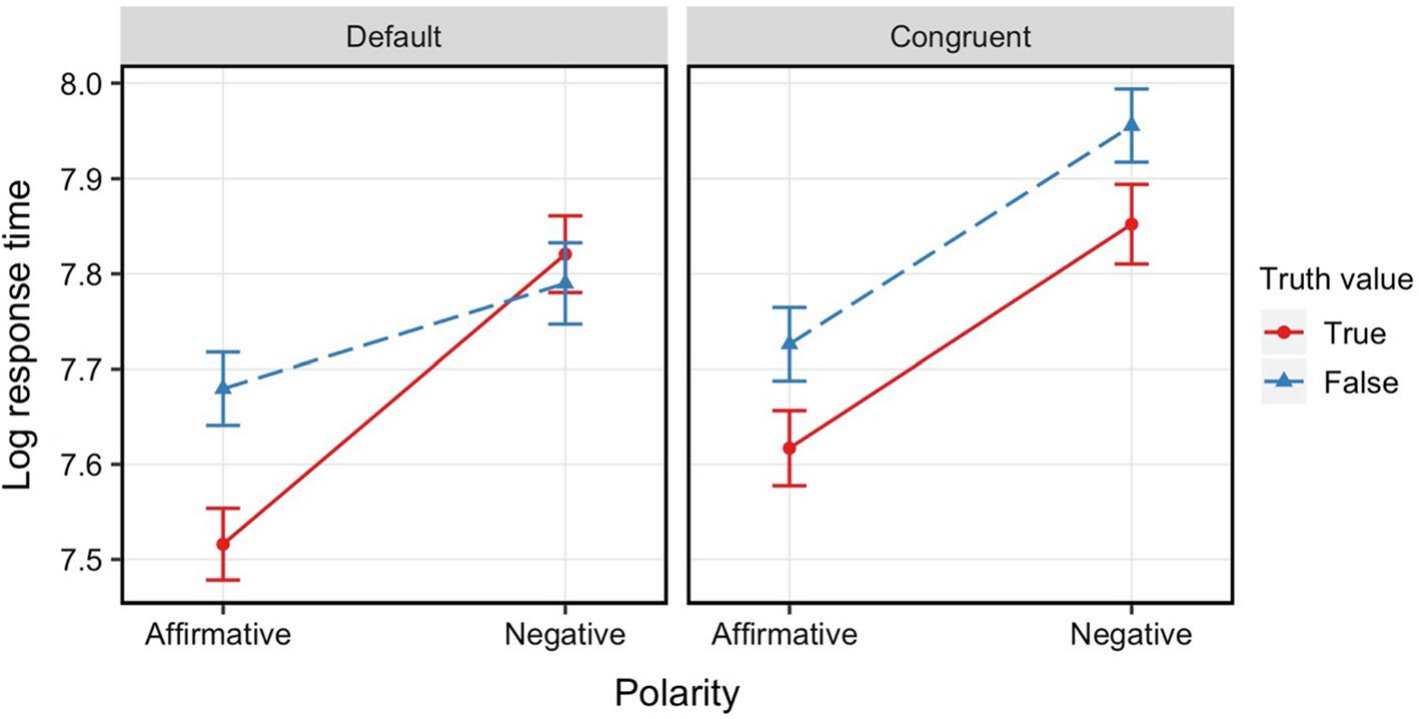
Mean reaction times for each condition and context in Experiment 1b. *Error bars* represent 95% confidence intervals

Figure 5 summarizes mean reaction times for each condition and context. We constructed a linear mixed effects regression model predicting ﻿logarithmised reaction times from context (default or congruent), polarity (affirmative or negative), truth value (true or false), and their interaction, including random effects structure as described before.^10^

The analysis showed main effects of polarity (β =  − 0.22, *SE* = 0.02, *p* < 0.001), truth value (β =  − 0.08, *SE* = 0.01, *p* < 0.001), and context (β =  − 0.09, *SE* = 0.01, *p* < 0.001): verifying negative sentences took longer than verifying positive sentences, ‘false’ responses took longer than ‘true’ responses, and responses took longer following a congruent question than following a default question. There was a significant interaction between polarity and truth value (β =  − 0.11, *SE* = 0.02, *p* < 0.001), and crucially, a significant three-way interaction (β =  − 0.19, *SE* = 0.04, *p* < 0.001). None of the other interactions was significant.

Follow-up analyses revealed that the default context showed an interactive pattern between polarity and truth value (β =  − 0.20, *SE* = 0.03, *p* < 0.001), whereas the congruent context showed only main effects (polarity: β =  − 0.23, *SE* = 0.02, *p* < 0.001; truth value: β =  − 0.10, *SE* = 0.02, *p* < 0.001). In the default context, participants took longer to correctly respond to FA compared to TA (TA < FA, β =  − 0.17, *SE* = 0.02, *p* < 0.001), however, no difference was found between TN and FN (*p* = 0.20. TN: *M* = 2704, *SD* = 1154; FN: *M* = 2658, *SD* = 1202).

### Cluster analyses

As predicted, we found a three-way interaction in Experiment 1b and this broke down as a polarity*truth-value interaction in the Default context, but only main effects in the Congruent context. Regarding the Default context result, previous research suggests that such an interaction arises from participants adopting a two-step procedure on at least some of the trials. As discussed above, some researchers have examined the responses of individuals in an experimental session and found that they can be grouped according to patterns of reaction time to response (MacLeod et al., 1978; Matthews et al., 1980). This work has attracted critical commentary on the grounds that similar response-time patterns among sub-groups of participants does not necessarily reflect similar strategies among the participants in those groups (Kaup et al., 2007a, 2007b; Marquer & Pereira, 1990). It seems that what is needed is some independent confirmation that groupings according to different RT patterns reflect different strategies. Thus a simple grouping analysis of the participants according to RT patterns in an SPV task may be of limited value. However, in our Experiment 1b, we have a study whose outcomes show that our participants’ response times differ depending on which context the sentences were presented in, and the differences are in line with our predictions. Our account also predicts that, if we examine the default context responses to see if any sub-groupings of individuals can be made based on RT patterns, then we would expect to find those participants who show a polarity*truth-value interaction will change their behaviour in the congruent context.

**Fig. 6:**
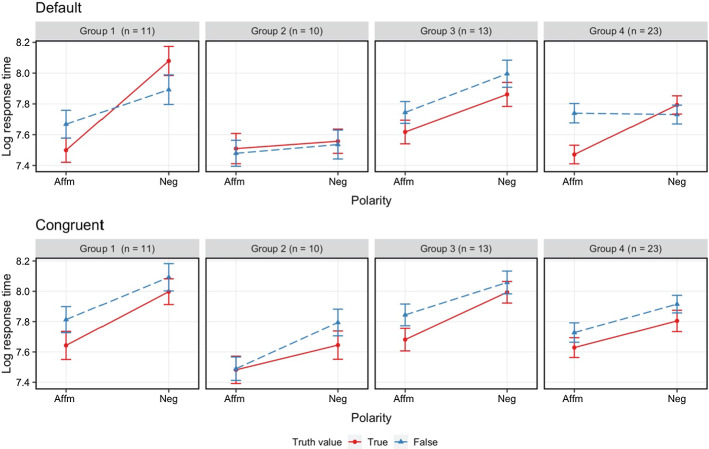
Experiment 1b, mean RT for each condition and group in two different contexts (above: default context; below: congruent context).* Error bars* represent 95% confidence intervals

To examine whether our participants fall into different groups based on their RT differences across the four Default-context conditions, we conducted a K-means clustering analysis (MacQueen, 1967). Typically, K-means analysis can partition a given dataset into a specific number of groups based on the Euclidean distance between the object and the centroid (for a review of data clustering and K-means see Jain, 2010). For this analysis,^11^ we first calculated four mean differences (i.e. difference between TA and FA, TN and FN, TA and TN, FA and FN) in the Default context for each participant. These input vectors are suggested by the overall analysis of the data, reflecting the interactive pattern found between polarity and truth-value. Then we estimated the optimal number of clusters in this four-dimensional dataset by computing total intra-cluster variation using different numbers of clusters. The comparisons suggested a four-cluster solution. Thus, we classified participants into four groups. K-means algorithm iteratively assigned observations to their closest centroids until the inter-group similarity was as low as possible. Once finished, we took K-means results and plotted the original dataset by group as shown in Fig. 6.

For each group in each context, we fitted a linear mixed effects regression model predicting ﻿logarithmised reaction times from polarity (affirmative or negative), truth value (true or false), and their interaction, including random intercepts for participants and items. Random slopes were not included in these models as they led to non-convergence or overfitting. Tables 3 and 4 summarized the results of the mixed models.

**Table 3:** For each group in the default context, fixed effect estimates for LMER of reaction times (log(RT) ~ Polarity * Truth value + ( 1 | Participant) + (1 | Item). The two-level factors Polarity (affirmative: 0.5, negative: –0.5) and Truth value (true: 0.5, false: –0.5) were deviation coded

	Group 1			Group 2			Group 3			Group 4		
*Predictors*	*β*	*SE*	*p*	*β*	*SE*	*p*	*β*	*SE*	*p*	*β*	*SE*	*p*
(Intercept)	7.79	0.08	** < 0.001**	7.53	0.08	** < 0.001**	7.80	0.06	** < 0.001**	7.69	0.06	** < 0.001**
Polarity	−0.41	0.04	** < 0.001**	−0.06	0.03	0.070	−0.24	0.04	** < 0.001**	−0.15	0.02	** < 0.001**
Truth value	0.01	0.04	0.754	0.03	0.04	0.456	−0.14	0.03	** < 0.001**	−0.11	0.02	** < 0.001**
Polarity: TV	−0.36	0.07	** < 0.001**	0.00	0.07	0.984	0.02	0.07	0.767	−0.34	0.04	** < 0.001**
N	11 _Participant_			10 _Participant_			13 _Participant_			23 _Participant_		
	64 _Item_			64 _Item_			64 _Item_			64 _Item_		
Observations	319			292			368			674		

*p* values < 0.05 are in bold

**Table 4:** For each group in the congruent context, fixed effect estimates for LMER of reaction times (log(RT) ~ Polarity * Truth value + ( 1 | Participant) + (1 | Item). Polarity and Truth value were deviation coded

	Group 1			Group 2			Group 3			Group 4		
*Predictors*	*β*	*SE*	*p*	*β*	*SE*	*p*	*β*	*SE*	*p*	*β*	*SE*	*p*
(Intercept)	7.90	0.07	** < 0.001**	7.60	0.08	** < 0.001**	7.90	0.05	** < 0.001**	7.78	0.07	** < 0.001**
Polarity	−0.29	0.04	** < 0.001**	−0.23	0.03	** < 0.001**	−0.26	0.03	** < 0.001**	−0.19	0.02	** < 0.001**
Truth value	−0.13	0.04	**0.002**	−0.08	0.03	**0.014**	−0.11	0.03	** < 0.001**	−0.09	0.02	** < 0.001**
Polarity: TV	−0.06	0.08	0.482	0.15	0.07	**0.028**	−0.09	0.07	0.146	−0.01	0.04	0.768
N	11 _Participant_			10 _Participant_			13 _Participant_			23 _Participant_		
	64 _Item_			64 _Item_			64 _Item_			64 _Item_		
Observations	298			303			370			667		

*p* values < 0.05 are in bold

34 participants (Groups 1, 4) showed the interactive pattern in the default context, but only main effects in the congruent context. 13 participants (Group 3) showed only significant main effects in both contexts. 10 participants (Group 2) showed a less clearly interpretable pattern, although this group did show a clear delay for False Negative relative to True in the Congruent condition.

The results of this cluster analysis add further support to our account of SPV tasks: A subset of participants generally take longer to respond True to negative sentences than False, when presented in a polar question context, but these same people take longer to respond False when the context is congruent. In fact, all groups in the optimal cluster analysis took longer on False negative items in the congruent context.

**Fig. 7:**
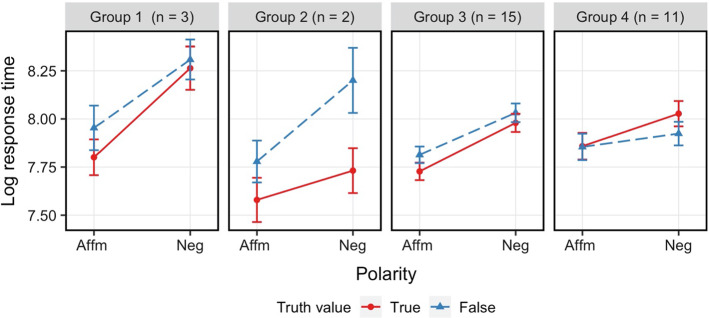
Experiment 1b, mean RT for each condition and group. *Error bars* represent 95% confidence intervals

It is instructive to consider a cluster analysis for our baseline study, Experiment 1a, in which no context is used. As shown in the analysis of Exp. 1a, the two main factors that affect the reaction time are polarity and truth value, and no interactive relation was found between the two factors. Hence, we clustered participants according to their RT differences on negative – affirmative and false – true. Again, we classified participants into four groups (the optimal number of clusters in the data). Figure 7 shows the RT pattern for each group.

As summarized in Table 5, only one third of the participants (Group 4) showed a trend for an interactive pattern, being the only group who took longer to respond True to negative sentences. The results for the cluster analysis of this baseline study are instructive about the effect of context in Experiment 1b, where we find a larger subset of participants (more than half) in a group that tend to employ the two-step procedure in polar question contexts. In congruent question contexts, by contrast, no groups show two-step behaviour.

**Table 5:** For each group fixed effect estimates for LMER of reaction times (log(RT) ~ Polarity * Truth value + ( 1 | Participant) + (1 | Item). Polarity and Truth value were deviation coded

	Group 1			Group 2			Group 3			Group 4		
*Predictors*	*β*	*SE*	*p*	*β*	*SE*	*p*	*β*	*SE*	*p*	*β*	*SE*	*p*
(Intercept)	8.08	0.16	** < 0.001**	7.82	0.05	** < 0.001**	7.89	0.05	** < 0.001**	7.93	0.09	** < 0.001**
Polarity	−0.41	0.05	** < 0.001**	−0.29	0.06	** < 0.001**	−0.24	0.02	** < 0.001**	−0.14	0.03	** < 0.001**
Truth value	−0.11	0.04	**0.004**	−0.33	0.06	** < 0.001**	−0.07	0.02	** < 0.001**	0.03	0.03	0.181
Polarity: TV	−0.11	0.08	0.147	0.26	0.11	**0.022**	−0.03	0.04	0.362	−0.10	0.05	0.054
N	3 _Participant_			2 _Participant_			15 _Participant_			11 _Participant_		
	64 _Item_			63 _Item_			64 _Item_			64 _Item_		
Observations	180			119			871			641		

*p* values < 0.05 are in bold

### Discussion

Results of Experiment 1b showed the predicted effect of question context on participant processing strategies. Positive polar questions, ‘Is the banana peeled?’ indicate a context where the positive argument of negation is considered a live possibility, while a wh-question with negative predicate ‘Which one isn’t peeled?’ suggests a context which presupposes that one thing is not peeled. Our account of the effects of negation in laboratory tasks is built on the idea that participants process the stimuli to infer a source of relevance as well as content and these processes can in some cases, but not all, draw attention to the positive argument of negation. Only in polar-question contexts does the data indicate that among some participants is there a tendency to complete the verification task in two steps. These same participants show a pattern consistent with the one-step procedure in congruent contexts, together with the other participants.

We can compare the results of Experiment 1b with those of Experiment 1a, where we saw only a little evidence overall of two-step procedure. That is, overall in Experiment 1a there was no significant polarity*truth-value interaction, but the cluster analysis revealed a grouping of participants for whom there was a marginal interaction. The results of each of the context conditions in Experiment 1b thus look somewhat different from this baseline set of results. This tells us that the polar question context highlighted further the relevance of the positive proposition, while the congruent context had an opposite effect. We attribute the absence of polarity*truth-value interaction overall in the baseline study in part to the fact that the predicates were naturally binary, and so the cost of inferring the actual state of affairs is lower than it is for items in other SPV studies. So, even if a participant may be liable to infer a positive context, it was relatively easy for them to ignore that state of affairs and simply evaluate the negative sentence against the picture. Moreover, Tian and Breheny (2016) speculate that the two-image stimuli may provide a weak cue to a context rather like our congruent context. This is because on each trial, each object is in the opposite state to the other (one open/one not open, one peeled/one not peeled, etc.). Thus if participants detect this (albeit weak) cue, they may develop a presupposition along these lines: given the property (e.g. *being peeled*) one object will have that property and one will be in the opposite state. They could thus understand the sentence, ‘the banana is not peeled’ to resolve the question of which object is in the opposite state – effectively like our congruent context. For this reason, we feel our two-picture stimuli may not as strongly suggest a default context as our polar question context in Experiment 1b and this is why we see evidence of greater use of two-step procedure in the default context of 1b than in the baseline.

## General Discussion

There are two plausible and well-supported traditions in research on psychological processes underlying the use of natural language negation. Across a range of paradigms, compelling evidence has been produced to suggest that when presented with a negative sentence, *[not[S]]*, participants actively represent the content of the argument *[S].* This has led to the view that the positive argument is represented as part of an overall comprehension process for negation. Strong versions of the Composite view hold that representations of the argument form a necessary part of negative sentence comprehension (Clark & Chase, 1972; Kaup et al, 2006). The second tradition in negation research stems from the idea that negation is often difficult to understand out of context (Horn, 1989; Nieuwland & Kuperberg, 2008; Wason, 1965). While this tradition has produced compelling evidence that well-contextualised negative sentences are not costly to process and often do not seem to require representing the positive argument, the contextualist tradition has not provided a clear account of why the positive is often actively used. According to our proposals, representations of the positive state of affairs emerge naturally when participants are presented with negative sentences because comprehension processes reflect the dual goals of inferring sentence context as well as sentence content. A critical component of sentence context is its Source of Relevance: the information state in which the speaker expects sentence content to achieve relevance. Because simple negative sentences are so frequently used when the positive is a live possibility in the discourse, probabilistic processes will take this to be the SoR unless other cues are available which override this default. Our conjecture has been that, when there is information available to comprehension processes which suggests a different kind of context, we do not detect as strong an effect of the positive. This was shown in previous work in probe recognition (Tian et al., 2010) and visual world (Tian et al., 2016) paradigms. In this paper, we developed sentence-picture verification (SPV) tasks to test our proposals and found clear support once again. In the following discussion, we will take up the question of how the proposals we advocate bear on the specific requirements of SPV tasks.

We take a broadly interpretivist picture of negative sentence comprehension processes. That is to say, we assume that comprehension processes are geared to compute inferences from an utterance on the assumption that what the speaker is saying is relevant and true. As has been observed before (Tanenhaus et al., 1976), verification tasks are metalinguistic tasks and the processes supporting verification, though frequent enough, are not necessarily typical of comprehension processes in everyday discourse. When asked to verify a negative sentence against a picture, participants have two verification procedures at their disposal because of the truth-functional semantics of negation. They can represent the argument of negation and consider if it should be rejected or not. To do this requires evaluating the positive but formulating a response whose polarity is the reverse of the evaluation (2-step). Alternatively, they can directly evaluate the negative sentence by inferring whether the state of affairs in the image is consistent with the truth of the sentence (1-step). Previous studies provide ample evidence that participants adopt both. Our view is that an important factor in determining which procedure is adopted is inferred context. When an experimental stimulus is absent of contextual information which would indicate a non-default context, participants’ attention is drawn to the positive state of affairs and this can make it more difficult to infer actual states of affairs against which to evaluate the image. Moreover, attention to the positive can facilitate adoption of the two-step procedure. By contrast, composite accounts explain the two-step procedure as a natural outcome of negative sentence processes which yield representations of the positive as part. Composite accounts have explained the use of one-step procedure in terms of the time between presentation of sentence and image (Clark & Chase, 1972; Kaup et al., 2007b), and/or participants adopting a task specific strategy to convert negative sentence to positive (Clark, 1974) or to an image (Matthews et al., 1980). The results of Experiment 1 show a clear effect of context on which strategies are adopted and so support our account. The results also provide a challenge for composite accounts since our stimuli do not delay presentation of the image. In addition, we see from the cluster analyses that the same participants who adopt a two-step procedure on Default context trials, adopt a one-step procedure on Congruent trials which are mixed in the same session.

We advocate an interpretivist approach to negative sentence processing, based on the truth-functional properties of negation and general language processing principles. We believe that adoption of our ideas about the role of Source of Relevance in comprehension processes obviate the need for strict composite approaches. Rather, we shed light on why apparently composite representations are constructed in the default case.

## Footnotes

1 This is an attribution made in Clark and Chase (1972), who also note similar ideas were formalised in models by Just and Carpenter (1971) and Trabasso, Rollins, and Shaughnessy (1971).

2 The source of relevance for an utterance is often described in terms of a ‘Question Under Discussion’ (QUD). Detailed proposals to define relevance in terms of QUDs have been outlined in Roberts (1996) and Ginzburg (2012). Here we adopt a more neutral idea, ‘Source of Relevance’ and remain uncommitted to whether relevance should be modelled in terms of questions—see Breheny (2019).

3 The experiment can be viewed at https://spellout.net/ibexexps/chao.sun/neg-noqud/experiment.html. Lists of items are available at https://github.com/LING-sunc/neg-noqud.git.

4 Prolific is a commercial crowdsourcing platform, available at https://www.prolific.co.

5 r-formula: glmer(Correctness ~ Polarity * TV + (Polarity * TV || subject) + (Polarity * TV || item), data, family = "binomial", control = glmerControl(optimizer = "bobyqa")). || indicates that the correlation between random intercept and random slopes was removed due to issues of convergence.

6 r-formular: lmer(logRT ~ Polarity * TV + (Polarity * TV || subject) + (Polarity * TV || item), data). We followed normal practive of taking logarithmised reaction times in our analysis as our RTs were not normally distributed. However, we repeated this analysis, as well as the reaction time analyses for Experiment 1b, using raw reaction times. The patterns in data did not differ across different dependent measures. Thus, here we only reported the analyses using log-transformed reactions times.

7 The experiment can be viewed at https://app.gorilla.sc/openmaterials/249101.

8 See Anwyl-Irvine, Massonnié, Flitton, Kirkham, & Evershed (2020) for a detailed description of Gorilla.

9 r-formula: glmer(Correctness ~ Context * Polarity * TV + (Context + Polarity + TV || subject) + (Context + Polarity + TV || item), data, family = "binomial", control = glmerControl(optimizer = "bobyqa")).

10 r-formular: lmer(logRT ~ Context * Polarity * TV + (Context + Polarity + TV || subject) + (Context + Polarity + TV || item), data).

11 Based on the model fit of a hypothesized linear regression, MacLeod et al. (1978) inferred that their participants fell into two groups that differentiate on strategy use. Similarly, we also aim to investigate participants’ cognitive strategies. However, we use K-means clustering in determining the groupings. K-means clustering is an unsupervised algorithm which makes inferences about the number of clusters using only input vectors. Hence our procedure uses fewer a priori assumptions. Our optimal number of clusters ensures that the homogeneity of each cluster is optimal (k-means minimize the within group variance and maximize the between group variance). In addition, we did not impose any hypothesized regression to our data, rather we re-examined the effect of independent variables within each group.
